# Prognostic significance of preoperative CT findings in patients with advanced gastric cancer who underwent curative gastrectomy

**DOI:** 10.1371/journal.pone.0202207

**Published:** 2018-08-09

**Authors:** Chae Jung Park, Nieun Seo, Woo Jin Hyung, Woong Sub Koom, Hyo Song Kim, Myeong-Jin Kim, Joon Seok Lim

**Affiliations:** 1 Department of Radiology, Research Institute of Radiological Science, Severance Hospital, Yonsei University College of Medicine, Seoul, Korea; 2 Department of Surgery, Severance Hospital, Yonsei University College of Medicine, Seoul, Korea; 3 Department of Radiation Oncology, Yonsei Cancer Center, Yonsei University College of Medicine, Seoul, Korea; 4 Division of Medical Oncology, Department of Internal Medicine, Yonsei Cancer Center, Yonsei University College of Medicine, Seoul, Korea; University Hospital Llandough, UNITED KINGDOM

## Abstract

**Background:**

Preoperative therapy has gained wide interest in advanced gastric cancer patients due to its potential advantages of improved disease control. Selection of high risk patients based on preoperative staging is crucial to choose the candidates for neoadjuvant therapy.

**Methods:**

Our institutional review board approved this retrospective study and waived the requirement for patient consent. We searched 394 advanced gastric cancer patients (pT2-4) who underwent curative resection in 2010 without neoadjuvant therapies. Two abdominal radiologists independently reviewed the preoperative CT including tumor depth on CT (CT-tumor depth), which was categorized as follows: intramural, minimal extramural(<1mm), spiculated extramural(≥1mm) and nodular extramural infiltration. The impact of clinicoradiologic factors on disease recurrence and disease free survival (DFS) was evaluated. Recursive partitioning analysis was performed to suggest prediction models for recurrence.

**Results:**

Of total 394 patients, 86 patients (21.8%) experienced recurrence. Spiculated (≥1mm) and nodular extramural tumor infiltration and CT size of 5-10cm were independent predictors of disease recurrence and significantly associated with worse DFS. Lymph node involvement on CT was not significantly associated with patient outcome. Among patients with same pT4a stage, the recurrence rate rises and DFS gets worse as the extramural tumor infiltration progresses (*P* < 0.001). The prediction model for recurrence revealed that size and CT-tumor depth were the two major discriminating factors.

**Conclusion:**

CT-tumor depth and size could be used as independent predictors for prognosis. Preoperative CT can be used for prognostic stratification to select high risk patients for whom neoadjuvant therapies might be considered.

## Introduction

Gastric cancer is the third and fifth leading worldwide cause of death in males and females, respectively [1] and is one of the most common cancers in Korea [2]. The only curative treatment for advanced gastric cancer is complete resection of tumors with negative margins (R0 resection) and D2 lymphadenectomy. However, a significant number of completely resected patients experience tumor recurrence [3, 4]. For locally advanced gastric cancer, perioperative chemotherapy has been established as the standard treatment to overcome high rates of recurrence [5, 6]. Recently, significant evidence has indicated the advantages of neoadjuvant therapies in patients with gastric cancer. Early treatment of distant microscopic disease and possible downstaging of the primary tumor which might be achieved by neoadjuvant therapies could yield a better outcome [7–9]. Several studies have reported that high R0 resection rate and survival were achieved with neoadjuvant chemotherapy followed by curative surgery [10–12].

In gastric cancer, computed tomography (CT) is the modality of choice for preoperative staging. To select candidates for neoadjuvant treatment, selection of high risk patients based on preoperative staging is needed. In cases of locally advanced rectal cancer, neoadjuvant chemoradiotherapy is selectively performed when high-risk findings for tumor recurrence (positive circumferential margin, extramural venous invasion, extramural tumor spread, etc.) are detected on preoperative magnetic resonance imaging (MRI) [13, 14]. However, limited data are available to stratify patients with advanced gastric cancer.

In this study, we aimed to investigate preoperative prognostic stratification based on preoperative CT findings in patients with advanced gastric cancer in order to select high-risk patients who might benefit from neoadjuvant therapy.

## Materials and methods

### Patient selection

This retrospective study was approved by institutional review board from our tertiary institution, Severance hospital, Yonsei University College of medicine. Requirement for informed consent was waived. After approval by the institutional review board, we retrospectively searched a total of 452 patients with advanced gastric cancer (pT2-4) who underwent curative surgery without neoadjuvant therapy in 2010. Patients who had double primary cancer (n = 17), histology other than adenocarcinoma (n = 1), less than 2 years of follow-up (n = 8), and history of previous endoscopic mucosal resection (n = 1) were excluded. Patients with insufficient preoperative CT images with slice thickness more than 5 mm were also excluded (n = 6). Thus, total 419 patients were analyzed. Demographic data (age and sex) were collected using electronic medical records.

### Protocol for CT

CT scans were performed with a 16- or 64-channel multidetector CT scanner (Somatom Sensation 16 and Sensation 64; Siemens Medical Solutions, Forchhein, Germany and Lightspeed VCT, GE Medical Systems, Milwaukee, WI, USA). Images were acquired from diaphragm level to the symphysis pubis with detector collimations of 16 x 0.75 mm or 64 x 0.6 mm. Other scanning parameters were as follows: 160 mAs; 120 kVp; table speed, 24 mm per rotation; and gantry rotation time, 0.5 seconds. For gastric distention, gas distention with 2 packs of effervescent granules was introduced. All patients received 120-150ml contrast medium intravenously using an automatic injector at a rate of 3–4 ml/s. Images of portal phases were obtained. Axial and coronal images were reconstructed with 3-mm-thick sections and a 3 mm interval.

### Image review

**Fig 1:**
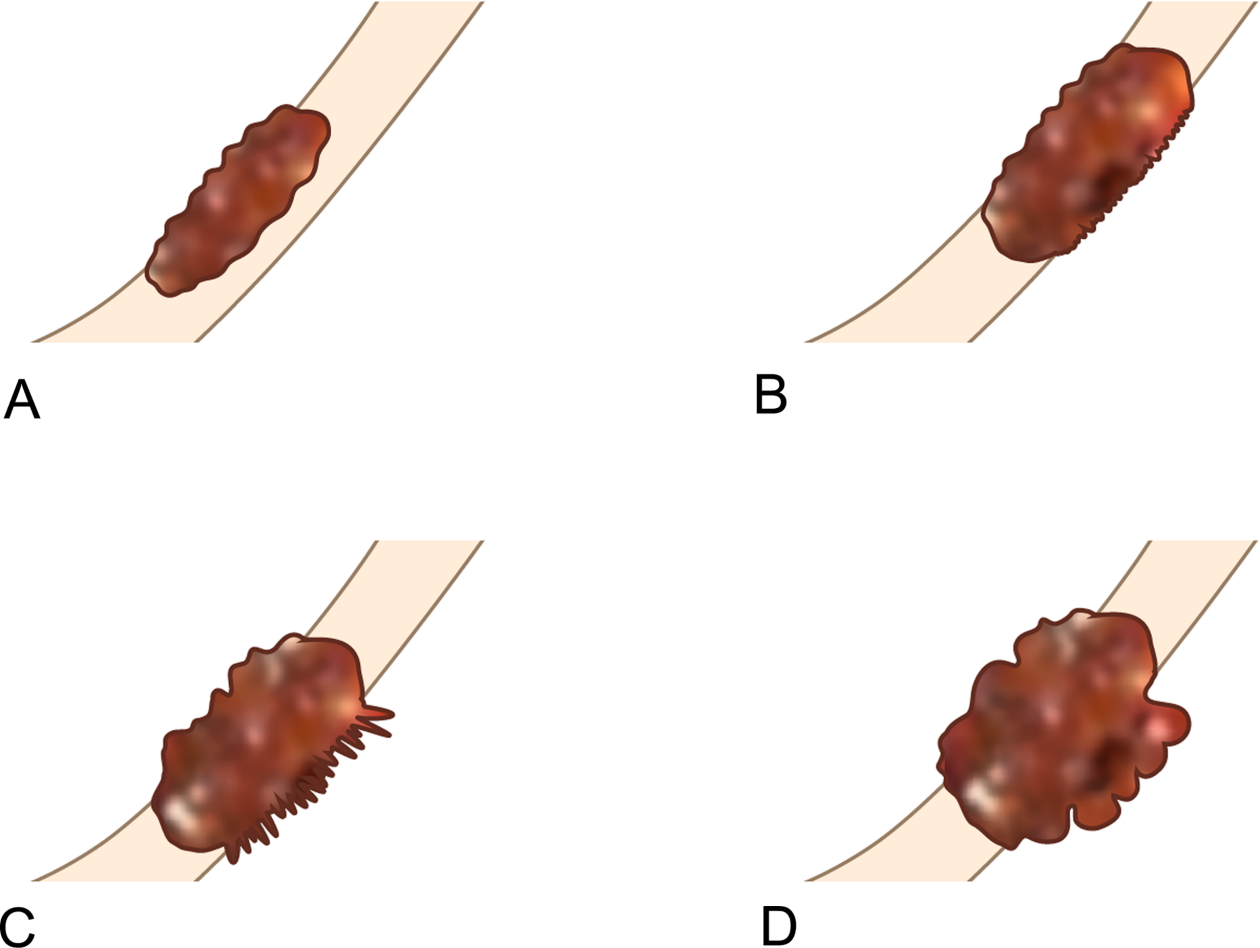
Schematic illustration showing four different groups of computed tomography (CT)-tumor depth. (A) Group 1, tumors confined to the stomach wall without extramural tumor infiltration. (B) Group 2, transmural involvement of the tumor with minimal extramural infiltration less than 1 mm. (C) Group 3, transmural involvement of the tumor with 1 mm or more spiculated extramural infiltration. (D) Group 4, transmural involvement of the tumor with nodular extramural infiltration.

**Fig 2:**
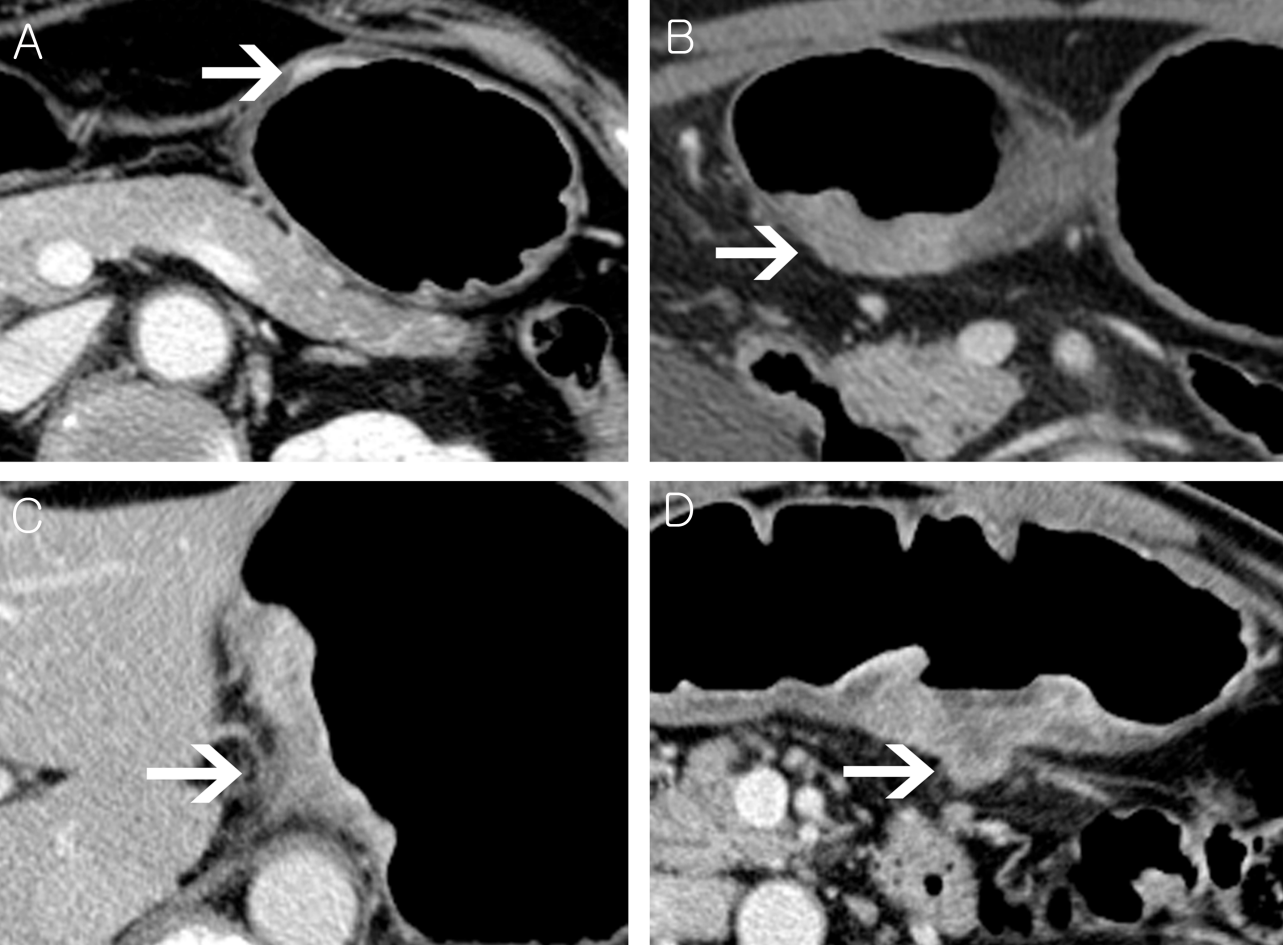
Axial images of contrast enhanced computed tomography (CT) demonstrating four different groups of CT-tumor depth. Group 1, 2, 3 and 4 in (A), (B), (C), and (D), respectively.

Two board-certified abdominal radiologists with more than 10 years of experience independently reviewed preoperative CT images and arrived at a consensus in cases with discrepancy. Both were blinded to pathologic reports and clinical outcomes. The analyzed CT imaging characteristics were tumor depth, lymph node (LN) status, presence of extramural vascular invasion (EMVI), tumor size, longitudinal extent, and Borrmann type. Tumor depth on CT (CT-tumor depth) was categorized into four groups. Group 1 was tumors confined to the stomach wall without extramural infiltration. Cases with extramural tumor infiltration were subdivided according to the degree of infiltration as follows: Group 2, transmural involvement of the tumor with minimal extramural infiltration less than 1 mm; group 3, transmural involvement of the tumor with 1 mm or more spiculated extramural infiltration; and group 4, transmural involvement of the tumor with nodular extramural infiltration (Figs 1 and 2). LN involvement on CT (CT-LN status) was categorized into two groups, N0-1 and N2-3, based on a previous study that reported that ≥ pN2 disease was diagnosed with a reasonably high sensitivity and specificity by CT [15]. Lymph nodes were considered metastatic if they had a short-axis diameter >8 mm. EMVI was identified as serpiginous extension of the tumor within a vascular structure. Tumor size was measured as the longest diameter on the axial or coronal plane. Tumor size was categorized into three groups: 1) less than 5 cm, 2) 5–10 cm, and 3) more than 10 cm. Longitudinal tumor extent was determined by an imaginary line from the gastroesophageal junction to the pyloric channel. Tumors which involved more than half of this line were classified as non-localized type and if not, those were classified as localized type. Tumors were analyzed according to the Borrmann classification and were classified as Borrmann type 4 in cases where infiltrative stomach cancer showed no definite ulceration or mass formation.

### Pathology

Postoperative pathologic stage was determined using the seventh edition of the International Union Against Cancer (UICC)/American Joint Committee on Cancer (AJCC) staging system. Slides were prepared from formalin-fixed, paraffin-embedded tissue blocks for histological examination. Hematoxylin–eosin (H–E)-stained slides of each tumor were reviewed to assess lymphovascular invasion (LVI), defined as the presence of tumor cell clusters or individual tumor cells within an endothelium-lined space lumen or destruction of a lymphovascular wall by tumor cells. At our institute, histopathologic reports contain only the presence or absence of LVI and do not specify whether it is vascular or lymphatic invasion. Therefore, we evaluated the diagnostic performance of CT-detected EMVI using pathologic LVI as the reference standard. Histologic grade was also retrieved from the pathologic report.

### Data analysis

The results of CT-tumor depth, CT-LN status, and EMVI on CT were compared with those of pT, pN staging, and pathologic LVI, respectively. The incidence of patients with pathologic serosal exposure among each subcategory of CT-tumor depth was calculated. Patients with pN2 or pN3 were considered to have ‘advanced nodal status.’ The incidence of patients with advanced nodal status was calculated for each subcategory of CT-tumor depth. The recurrence rate and DFS were evaluated according to CT-tumor depth in patients with the same pT4a stage.

### Statistical analysis

Statistical analysis was performed using SPSS 20.0.0 (SPSS, Chicago, IL) and R software (version 3.2.2; R Development Core Team, Vienna, Austria). Weighted kappa statistics were used to evaluate the interobserver agreement for CT-tumor depth. Cochran-Mantel-Haenszel tests were performed to examine the linear trend between extent of CT-tumor depth and pathologic LN status. Univariate associations of clinicoradiologic factors such as age, sex, and CT findings with recurrence status were assessed using chi-square or Fisher’s exact test. Subsequently, parameters with a *P* value less than 0.05 were included in multivariate logistic regression and the association of each parameter with recurrence was expressed as an odds ratio (OR) with a 95% confidence interval (CI).

Disease-free survival (DFS) after surgery was estimated from the date of operation to the date of recurrence or death using the Kaplan-Meier method and compared using the log-rank test. The impact of clinicoradiologic factors such as age, sex, and CT findings on DFS was evaluated using the multivariable Cox regression model. All clinicoradiologic variables except sex were entered into the multivariate model. Hazard radios (HR) and 95% CIs were generated.

On the basis of the results of logistic regression analysis for disease recurrence, recursive partitioning analysis (RPA) was performed to suggest prediction models for recurrence. Receiver operating characteristic (ROC) analysis was used to assess the discriminatory powers of the diagnostic tree model. The areas under the curve (AUCs) for receiver operating characteristic analysis were calculated.

All tests were 2-sided and *P* values less than 0.05 were considered statistically significant.

## Results

### Patient characteristics

After retrospective image analysis of enrolled 419 patients, 25 who had no identifiable stomach cancer at preoperative CT were excluded. Characteristics of 394 patients are summarized in Table 1. The median age of enrolled patients was 57 years (range, 23–86 years). Regarding pT stage, pT3-4 accounted for 74.4% (n = 293), while pT2 accounted for 25.6% (n = 101) of cases. TNM stage III patients represented 49.7% (196/394) of the total sample. Pathologic LVI was positive in 46.4% (183/394).Among total 394 patients, 82.5% (325/394) underwent adjuvant chemotherapy.

**Table 1:** Patient characteristics of included 394 patients.

Characteristics	Number	%
Sex		
Male	265	67.3
Female	129	32.7
Age (years), median (range)	57 (23–86)	
T classification^a^		
T2	101	25.6
T3	121	30.7
T4a	168	42.6
T4b	4	1.0
N classification^a^		
N0	141	35.8
N1	74	18.8
N2	79	20.1
N3	100	25.4
TNM stage^a^		
I IB	57	14.5
II		
IIA	69	17.5
IIB	72	18.3
III		
IIIA	61	15.5
IIIB	64	16.2
IIIC	71	18.0
Pathologic lymphovascular invasion		
No	211	53.6
Yes	183	46.4
Histologic grade		
Well/Moderate differentiation	135	34.3
Poor/signet ring cell/mucinous	259	65.7

UICC/AJCC, International Union Against Cancer/American Joint Committee on Cancer (seventh edition).

^a^UICC/AJCC staging system, seventh edition.

### Preoperative CT findings compared with pathologic data

The weighted kappa value between the two radiologists showed moderate agreement (0.555) for assessing the CT-tumor depth. The distribution of pT stage was as follows: pT2, n = 101; pT3, n = 121; pT4a, n = 168; pT4b, n = 4 (S1 Table). The incidence of pathologic serosal exposure among each subcategory of CT-tumor depth was as follows: intramural, 17.5%; minimal extramural (<1 mm), 44.7%; spiculated extramural (≥1 mm), 53.3%; and nodular extramural, 77.2% (Table 2).

**Table 2:** Incidence of patients with pathologic serosal exposure and advanced nodal status among each subcategory of CT-tumor depth.

CT-tumor depth	Patients with pathologic serosal exposure^a^ (%)	Patients with advanced nodal status^b^ (%)
Intramural	17.5% (22/126)	48.4% (61/126)
Minimal extramural (<1mm)	44.7% (34/76)	57.9% (44/76)
Spiculated extramural (≥1mm)	53.3% (72/135)	72.6% (98/135)
Nodular extramural	77.2% (44/57)	87.7% (50/57)

^a^Cases with pathologic T4a or T4b were considered to have serosal exposure.

^b^Advanced nodal status means pathologic N2 or N3 status.

The distribution of pN stage was as follows: pN0, n = 141; pN1, n = 74; pN2, n = 79; pN3, n = 100 (S2 Table). The sensitivity, specificity, and accuracy of CT-LN status for diagnosing advanced nodal status (pN2 and pN3) were 35.2%, 90.7%, and 65.5%, respectively.

As CT-tumor depth progressed, the incidence of patients with advanced nodal status (pN2-3) increased (intramural, minimal extramural (<1 mm), spiculated extramural (≥1 mm), and nodular extramural: 48.4, 57.9, 72.6, and 87.72%, respectively; *P* value < 0.001, Table 2). The average number of pathologic metastatic LNs also showed an increasing trend as CT-tumor depth progressed, as follows: tumors with intramural, minimal extramural (<1 mm), spiculated extramural, (≥1 mm) and nodular extramural infiltration showed 1.8, 3.5, 6.7, and 11.1 pathologic metastatic LNs, respectively.

The sensitivity, specificity, and accuracy of CT-detected EMVI for diagnosing pathologic LVI were 28.4%, 81.5%, and 56.9%, respectively.

The distribution of tumor size based on CT was as follows: less than 5 cm, 66.5% (n = 262); 5–10 cm, 28.2% (n = 111); and more than 10 cm, 5.3% (n = 21). The distribution of longitudinal extent of tumor was as follows: localized, 93.9% (370/394) and non-localized, 6.1% (24/394). Thirty-three patients were considered Borrmann type 4 (8.4%).

### Multivariate analysis of disease recurrence

The types of recurrence were classified as locoregional, peritoneal, or distant metastasis. Locoregional recurrence included a soft tissue mass at the gastric bed, upper retroperitoneal LN enlargement, or anastomosis site recurrence. Of 394 total patients, 86 (21.8%) experienced recurrence. Distant metastasis by hematogenous spread was the most frequent site of recurrence (59.3%, 51/86), followed by peritoneal recurrence (44.1%, 30/68) and locoregional recurrence (10.5%, 9/86). The liver was the most frequently involved site of recurrence (18.6%, 16/86). Majority had a single site of recurrence, while 12 patients (14.0%) had multiple sites of recurrence.

On univariate analysis, all radiologic variables were significantly associated with recurrence. Multivariate analysis with adjustment for age and CT size revealed that spiculated (≥1 mm) and nodular extramural tumor infiltration and CT size of 5–10 cm were independent predictors of disease recurrence (Table 3).

**Table 3:** Multivariable regression analysis of disease recurrence.

Variable	Univariate			Multivariate		
	OR	95% CI	*P*	OR	95% CI	*P*
CT-tumor depth (intramural)						
Minimal extramural(<1mm)	1.96	0.79–4.87	0.146	0.62	0.64–4.1	0.309
Spiculated extramural	4.38	2.07–8.26	<0.001	2.87	1.26–6.54	0.012
Nodular extramural	11.20	4.89–25.66	<0.001	6.66	2.64–16.8	<0.001
CT-LN status (cN0-1)						
cN2-3	2.97	1.76–5.08	<0.001	1.39	0.73–2.64	0.312
CT EMVI (Negative)						
Positive	3.81	2.27–6.40	<0.001	1.38	0.70–2.71	0.349
CT size (<5cm)						
5-10cm	1.	2.25–6.42	<0.001	2.04	1.12–3.73	0.021
>10cm	5.90	2.33–14.91	<0.001	2.7	0.99–7.36	0.052
Extent (localized)						
Non-localized	4.81	2.07–11.17	<0.001	1.69	0.53–5.40	0.375
Borrmann Type 4 (No)						
Yes	4.81	2.07–11.17	<0.001	1.75	0.64–4.76	0.277

OR, odds ratio; CI, confidence interval; EMVI, extramural vascular invasion.

**Fig 3:**
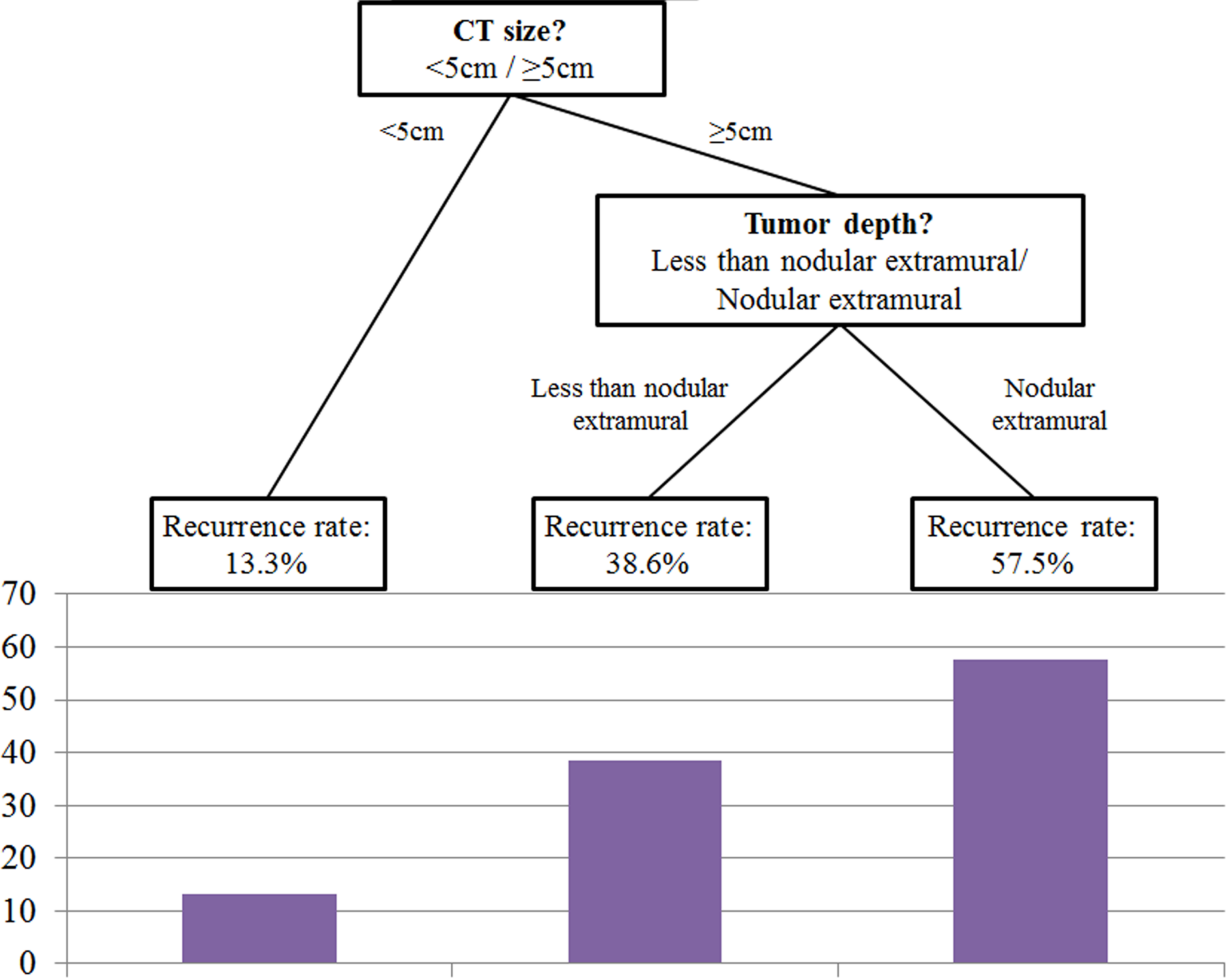
Prediction tree model for disease recurrence using recursive-partitioning analysis. Preoperative tumor size and CT-tumor depth were the two major discriminating factors for predicting disease recurrence.

Using recursive-partitioning analysis based on the classification and regression tree model, a prediction tree model for disease recurrence was established (Fig 3). Preoperative tumor size and CT-tumor depth were the two major discriminating factors for predicting disease recurrence. The sensitivity, specificity, and accuracy of this model were 51.2%, 96.1%, and 78.6%, respectively. ROC curve analyses revealed an AUC value of 0.736.

### Disease-free survival after curative surgery

**Fig 4:**
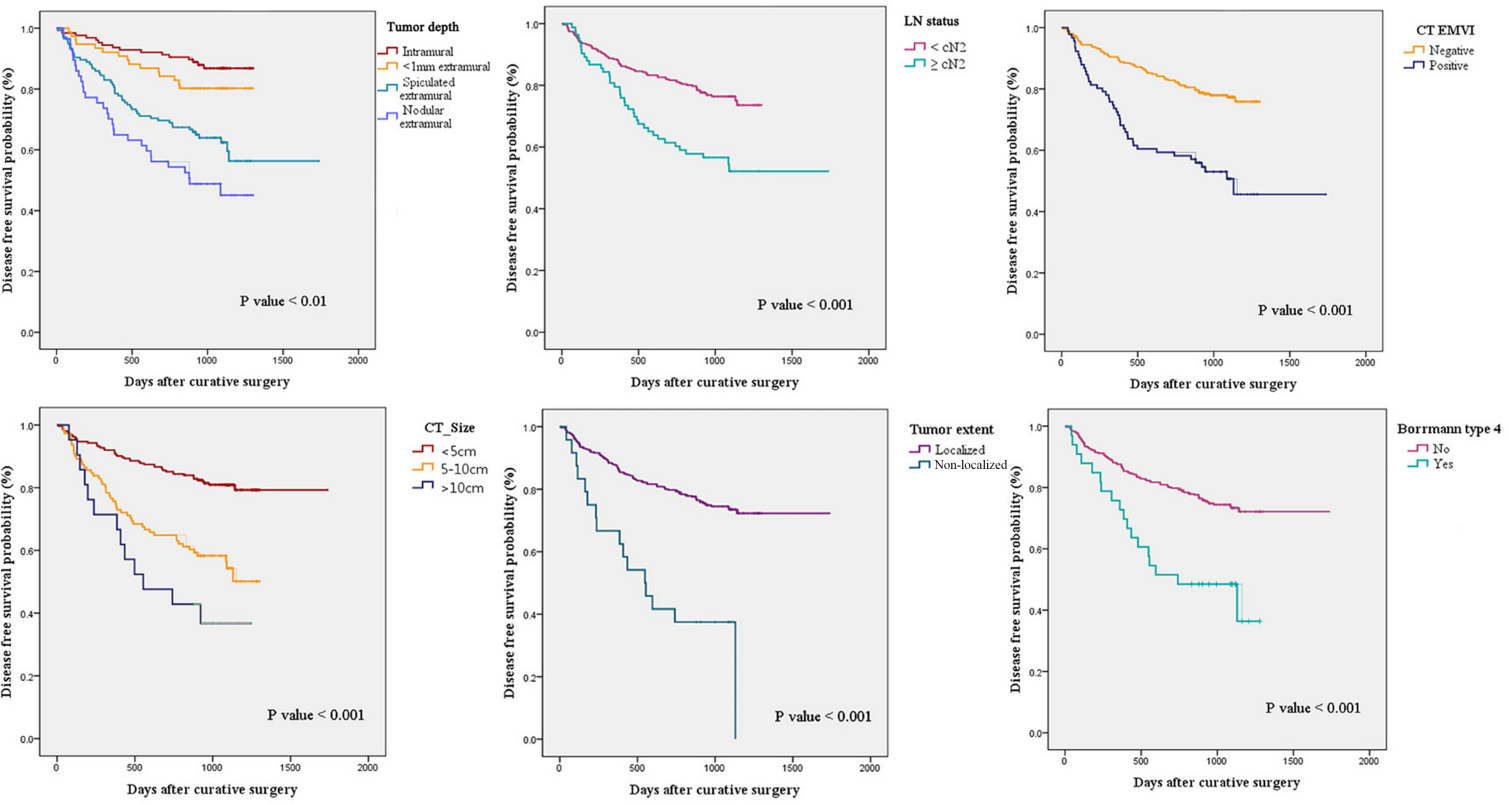
Kaplan-Meyer curves for disease free survival stratified to variable radiologic parameters. DFS was significantly worse in patients with CT findings of more advanced CT-tumor depth, CT-LN status, CT-detected EMVI, large size, non-localized longitudinal extent, and Borrmann type 4.

The median DFS after curative surgery was 44.9 months (95% CI 42.8–47.0 months). As shown in Fig 4, DFS was significantly worse in patients with CT findings of more advanced CT-tumor depth, CT-LN status, CT-detected EMVI, large size, non-localized longitudinal extent, and Borrmann type 4 (all *P* < 0.001).

Multivariate Cox regression model with adjustment of age and CT size revealed that spiculated or nodular extramural tumor infiltration, CT size of 5–10 cm, and non-localized tumor involvement of the stomach were significantly associated with worse DFS (Table 4).

**Table 4:** Multivariate cox regression model of disease free survival using clinicoradiologic factors.

Variable	HR	95% CI	*P*
Age (continuous)	1.02	1.00–1.03	0.086
CT-tumor depth (intramural)			
Minimal extramural(<1mm)	1.59	0.66–3.8	0.298
Spiculated extramural(≥1mm)	2.75	1.28–5.91	0.009
Nodular extramural	5.32	2.38–11.89	<0.001
CT-LN status (cN0-1)			
cN2-3	1.23	0.79–1.93	0.360
CT EMVI (Negative)			
Positive	1.19	0.73–1.92	0.490
CT size (<5cm)			
5-10cm	1.81	1.08–3.03	0.025
>10cm	1.67	0.7–3.97	0.248
Extent (localized)			
Non-localized	2.00	1.00–4.00	0.0498
Borrmann Type 4 (No)			
Yes	1.32	0.69–2.52	0.409

HR, hazard ratio; CI, confidence interval; EMVI, extramural vascular invasion.

### Subgroup analysis among patients with pT4a

**Fig 5:**
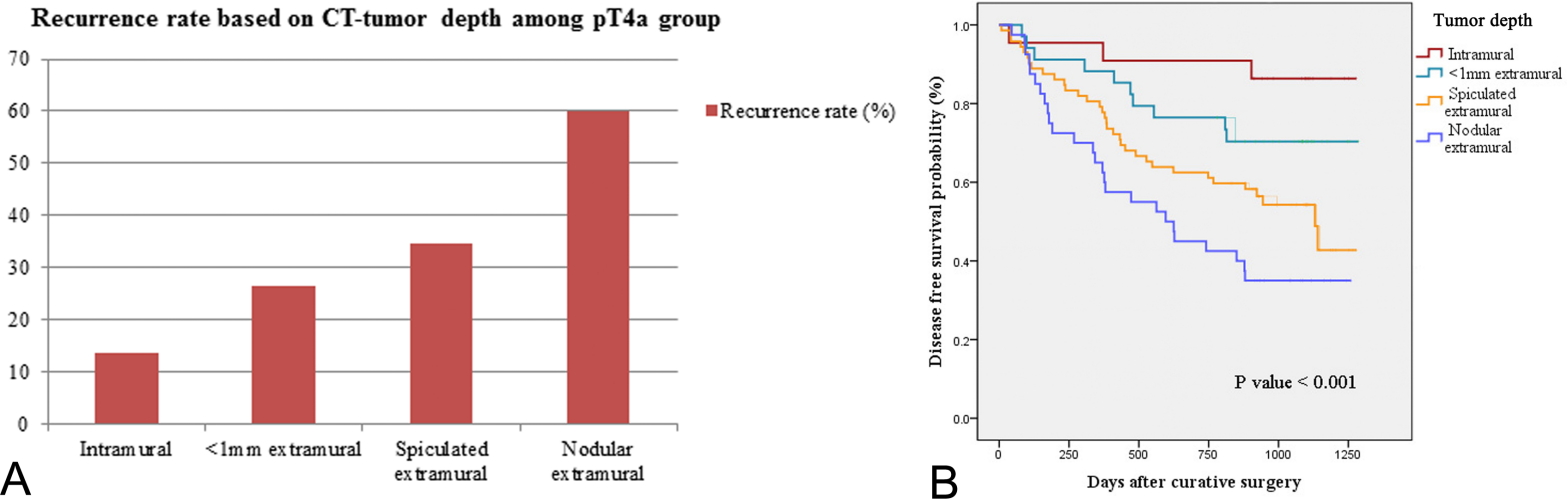
Subgroup analysis among patients with pT4a stage. As extramural tumor infiltration progressed, recurrence rate significantly increased (A) and the disease free survival became worse (B).

The recurrence rate in each category of CT-tumor depth among patients with pT4a was as follows: intramural, minimal extramural (<1 mm), spiculated extramural (≥1 mm), and nodular extramural showed rates of 13.6% (3/22), 26.5% (9/34), 34.7% (25/72), and 60.0% (24/40), respectively. As extramural tumor infiltration became more prominent, the recurrence rate increased with statistical significance (*P* < 0.001). DFS was also significantly worse in patients with more progressed extramural tumor infiltration among patients with same pT4a stage (*P* < 0.001) (Fig 5).

## Discussion

As neoadjuvant chemotherapy has emerged as an attractive treatment option in patients with advanced gastric cancer, CT staging prior to treatment has become more important for predicting prognosis. Traditionally, pathologic stage is the most important prognostic factor in gastric cancer. However, pathologic stage can only be determined after resection and preoperative treatment could alter the baseline pathologic stage. Effective and accurate preoperative staging is required in the era of preoperative therapy for resectable gastric cancer.

A large-scale retrospective study suggested that preoperative CT staging is an independent predictor of long-term survival and it should be regarded as a stratification factor in a randomized clinical trial of preoperative therapy in patients with gastric cancer [8]. However, their prognostic model was based on a study population including a considerable proportion of patients with lower clinical T stage (mucosa and submucosa). These early gastric cancer patients were excluded from the current study because they do not require neoadjuvant treatment. By selecting only advanced gastric cancer patients, the potential targets for neoadjuvant therapy became our study subjects and we aimed to identify patients who might benefit from neoadjuvant therapy.

The current seventh edition of the UICC/AJCC system separates tumors with extramural fat infiltration into T3 (subserosa invasion) and T4a (serosal exposure). This makes CT based T classification more challenging, because T3 and T4a lesions have similar CT findings (extramural soft tissue infiltration), and their differentiation is very difficult due to limitations of CT spatial resolution. For these reasons, we divided tumors as those with or without extramural infiltration. Tumors with extramural fat infiltration were classified into three subcategories according to the degree of extramural fat infiltration (minimal extramural (<1 mm), spiculated extramural (≥1 mm), and nodular extramural), instead of cT staging, which corresponds to pathological T staging. Our results demonstrated that tumors with more advanced extramural infiltration showed higher rates of pathologic serosal exposure. Also, tumors with spiculated (≥1 mm) and nodular extramural infiltration on CT indicate higher risk of recurrence and worse DFS compared to tumors with lesser extramural infiltration. Similar results have occurred in patients with the same pT4a stage, indicating that even among patients sharing the same pathologic T stage, prognosis can differ based on preoperative extramural tumor depth. The worse prognosis of tumors with spiculated (≥1 mm) or nodular extramural infiltration might be explained by higher rates of pathologic serosal exposure. In addition, more frequent LN involvement and a larger number of metastatic LNs have been observed in tumors with advanced extramural infiltration. This contributes to their worse prognosis because lymph node involvement is the most important factor for overall survival in patients with gastric cancer following curative resection and the survival rates markedly decrease with an increase in the number of metastatic lymph nodes [16, 17]. Therefore, we believe that our CT-tumor depth could be more easily assessed than T classification and might be used to stratify high-risk patients who might benefit from neoadjuvant therapies.

Pathologic N stage is one of the most reliable prognostic indicators for patients with resectable gastric cancer [17, 18]. However, accurate preoperative N staging by CT is limited because differentiation of small LNs with micrometastasis and large reactive LNs is difficult based on size criteria [8]. In our study, we categorized preoperative LN status into two groups (N0-1 vs. N2-3) based on a previous report that contrast-enhanced CT offers reasonably high sensitivity and specificity for ≥pN2 [15]. However, LN status on preoperative CT was significantly associated with disease recurrence and DFS only on univariate analysis, but not on multivariate analysis. This result could be attributed to inaccurate preoperative N staging by CT.

Pathological studies show that vascular invasion of gastrointestinal cancer allows tumor cells to embolize through the portal circulation, resulting in distant metastases through hematogenous spread [19]. MRI-detected EMVI in patients with rectal cancer is an established independent significant risk factor for poor prognosis and is used to select patients for neoadjuvant therapies [14, 19]. A previous pathological study demonstrated that EMVI was an independent pathologic feature for subsequent visceral metastases and worse disease-specific survival in patients with esophageal and gastric cancer [20, 21]. Recently, there was a study which assessed the clinical significance of preoperative CT-detected EMVI. Although the study provided a relatively short follow up period and small sample size, it suggested that CT-detected EMVI-positive patients had significantly lower 1-year progression free survival (PFS) than EMVI-negative patients, in addition, EMVI was also an independent prognostic factor in stage III gastric cancer [22]. According to our study, the CT-detected EMVI-positive group had a significantly worse 5-year DFS than the CT-detected EMVI-negative group. CT-detected EMVI showed a significant association with disease recurrence and DFS by univariate analysis, although it was not an independent factor by multivariate analysis. Thus, CT-detected EMVI might be used as a prognostic factor to stratify patients as high-risk who could undergo neoadjuvant therapy followed by surgery, as MRI-detected EMVI is used in rectal cancer. Until now, limited studies have addressed the clinical significance of CT-detected EMVI, and further studies are required for validation.

Tumors of 5–10 cm had significantly worse prognosis than tumors <5 cm. Tumors larger than 10 cm showed high odds ratio and hazard ratio for disease recurrence and DFS, respectively, but without statistical significance. This might be attributed to the small number of tumors larger than 10 cm (n = 21). Still, our study suggests that CT size is an independent prognostic factor and one of the major discriminators of the prognosis prediction model. A previously mentioned large retrospective study showed that tumor size ≥ 4.5 cm was significantly associated with worse overall survival in patients who underwent curative gastrectomy [8]. Tumor size was also one of the independent risk factors for disease recurrence among patients with T1-2 and lymph node-negative stomach cancer in the United States and China [23]. Additionally, macroscopic tumor size (≥7 cm) was one of the most important risk factors for peritoneal recurrence in patients with advanced stomach cancer who underwent adjuvant chemotherapy after D2 gastrectomy [24]. These results implicate large tumor size as a risk factor for poor prognosis in patients with stomach cancer.

Our study has some limitations. First, this is a retrospective study. Inclusion of patients with tumor stage pT2-4 who did not undergo neoadjuvant therapy might have led to selection bias. Second, preoperative staging was based on CT alone, which is not the best modality for staging T classification and peritoneal carcinomatosis. Although we assessed CT-tumor depth instead of classical cT staging for better characterization, we did not consider endoscopic ultrasound (EUS) findings, which provide better resolution compared to CT. Thus, preoperative staging using EUS might be used for validating our preoperative staging using CT-tumor depth. However, despite the favorable performance of EUS in staging, the influence of EUS on patient management remains controversial. EUS seems to have a greater impact on management of early stages rather than in advanced stages, thus, its role has been more emphasized in patients with early gastric cancer [25]. In addition, EUS is not routinely performed in all patients with advanced gastric cancer while CT, which is the modality of choice for preoperative staging, is more feasible and available in majority of patients. Thus, we believe that preoperative staging using CT itself without EUS conveys significance for assessing the tumor characterization.

In conclusion, as neoadjuvant therapies in patients with advanced gastric cancer have gained wide interest, clinical staging prior to preoperative treatment has become more important for predicting prognosis. CT-tumor depth and CT size could be used as independent predictors for prognosis. Tumors with extramural infiltration more than 1 mm showed significantly higher disease recurrence and worse DFS. CT can be used for prognostic stratification to select high risk patients for whom neoadjuvant therapies might be considered.

## Supporting information

S1 Table The distribution of pathologic T staging according to CT-tumor depth. (DOCX)

S2 Table The distribution of pathologic N staging according to CT-LN status by UICC/AJCC staging system. (DOCX)
