# Supplementary material for: Prognostic significance of preoperative CT findings in patients with advanced gastric cancer who underwent curative gastrectomy
Source: PLoS One. 2018 Aug 9;13(8):e0202207. doi: 10.1371/journal.pone.0202207 (PMC6084995; doi:10.1371/journal.pone.0202207)
Supplement: S1 Table — (DOCX) [file pone.0202207.s001.docx]

**Table S1. The Distribution of Pathologic T staging according to CT-tumor depth**

|  | Pathologic stage by UICC/AJCC^*^ | | | | |
| --- | --- | --- | --- | --- | --- |
| CT-tumor depth | pT2 | pT3 | pT4a | pT4b | Total |
| Intramural | 71 | 33 | 22 | 0 | 126 |
| Minimal extramural (<1mm) | 18 | 24 | 34 | 0 | 76 |
| Spiculated extramural (≥1mm) | 12 | 51 | 72 | 0 | 135 |
| Nodular extramural | 0 | 13 | 40 | 4 | 57 |
| Total | 101 | 121 | 168 | 4 | 394 |

^*^Values are number of patients.

UICC/AJCC, International Union Against Cancer/American Joint Committee on Cancer (seventh edition).
