# Supplementary material for: Prognostic significance of preoperative CT findings in patients with advanced gastric cancer who underwent curative gastrectomy
Source: PLoS One. 2018 Aug 9;13(8):e0202207. doi: 10.1371/journal.pone.0202207 (PMC6084995; doi:10.1371/journal.pone.0202207)
Supplement: S2 Table — (DOCX) [file pone.0202207.s002.docx]

**Table S2. The Distribution of Pathologic N staging according to CT-LN status by UICC/AJCC Staging System**

|  | Pathologic stage^*^ | | | | |
| --- | --- | --- | --- | --- | --- |
| CT-LN status | pN0 | pN1 | pN2 | pN3 | Total |
| cN0-1 | 128 | 67 | 58 | 58 | 311 |
| cN2-3 | 13 | 7 | 21 | 42 | 83 |
| Total | 141 | 74 | 79 | 100 | 394 |

^*^Values are number of patients.

UICC/AJCC, International Union Against Cancer/American Joint Committee on Cancer (seventh edition).
